# Sodium tanshinone IIA sulfonate stimulated Cl^−^ secretion in mouse trachea

**DOI:** 10.1371/journal.pone.0178226

**Published:** 2017-05-22

**Authors:** Peng-Xiao Chen, Yi-Lin Zhang, Jia-Wen Xu, Ming-Hao Yu, Jie-Hong Huang, Lei Zhao, Wen-Liang Zhou

**Affiliations:** 1 School of Life Sciences, Sun Yat-sen University, Guangzhou, Guangdong Province, China; 2 Department of Physiology, School of Basic Science, Guangzhou Medical University, Guangzhou, Guangdong Province, China; Forschungszentrum Borstel Leibniz-Zentrum fur Medizin und Biowissenschaften, GERMANY

## Abstract

Sodium tanshinone IIA sulfonate (STS) is a derivate of tanshinone IIA, a lipophilic compound in *Salvia miltiorrhiza*. This study aimed to investigate the effect of STS on ion transport in mouse tracheal epithelium and the mechanisms underlying it. Short-circuit current (*I*_sc_) was measured to evaluate the effect of STS on transepithelial ion transport. Intracellular Ca^2+^ imaging was performed to observe intracellular Ca^2+^ concentration ([Ca^2+^]_i_) changes induced by STS in primary cultured mouse tracheal epithelial cells. Results showed that the apical application of STS at mouse trachea elicited an increase of *I*_sc_, which was abrogated by atropine, an antagonist of muscarinic acetylcholine receptor (mAChR). By removing ambient Cl^−^ or applying blockers of Ca^2+^-activated Cl^−^ channel (CaCC), the response of STS-induced *I*_sc_ was suppressed. Moreover, STS elevated the [Ca^2+^]_i_ in mouse tracheal epithelial cells. As a result, STS stimulated Cl^−^ secretion in mouse tracheal epithelium via CaCC in an mAChR-dependent way. Due to the critical role of Cl^−^ secretion in airway hydration, our findings suggested that STS may be used to ameliorate the airway dehydration symptom in cystic fibrosis (CF) and chronic obstructive pulmonary disease (COPD).

## Introduction

Airway surface liquid (ASL) is a film of fluid lining on the apical surface of airway, consisting of a periciliary liquid layer and a mucus layer [1]. The presence of ASL is crucial for accelerating mucociliary clearance, since airway cilia beat in the periciliary liquid layer in low-viscosity to remove the particles and pathogens that are trapped in a mucus layer out of the airway. In addition, the height of periciliary liquid layer is reportedly fine-tuned by transepithelial Cl^−^ secretion and Na^+^ absorption, which promotes water movement by local concentration gradients [2]. The main Cl^−^ channels, located at the apical side of airway epithelial cells, are cystic fibrosis transmembrane conductance regulator (CFTR) and Ca^2+^ activated Cl^−^ channel (CaCC), both of which play crucial roles in mediating transepithelial Cl^−^ secretion [3]. Notably, cystic fibrosis (CF), an autosomal recessive disease caused by CFTR gene mutation, results in the dehydration of periciliary liquid layer and deteriorates mucus transport in airway, which is associated with the defective host defense and chronic bacterial infection in CF patients [4]. Apart from CF, other chronic airway inflammatory diseases such as COPD [5, 6], chronic bronchitis [7], and chronic rhinosinusitis [8], also have the same symptom of insufficient ASL secretion due to ion channels dysfunction.

The recent studies on several Chinese traditional medicines and their extracts, such as quercetin, resveratrol and *Cordyceps militaris*is, indicate that these substances have significant effects on ASL secretion by modifying ion channel activity in airway epithelium [9–11]. Nonetheless, the potential bio-safety problem owing to the complex components of Chinese medicine extracts limited their clinical application. Therefore, it is necessary to study the mechanisms of Chinese herbal monomer functions on ASL secretion.

**Fig 1:**
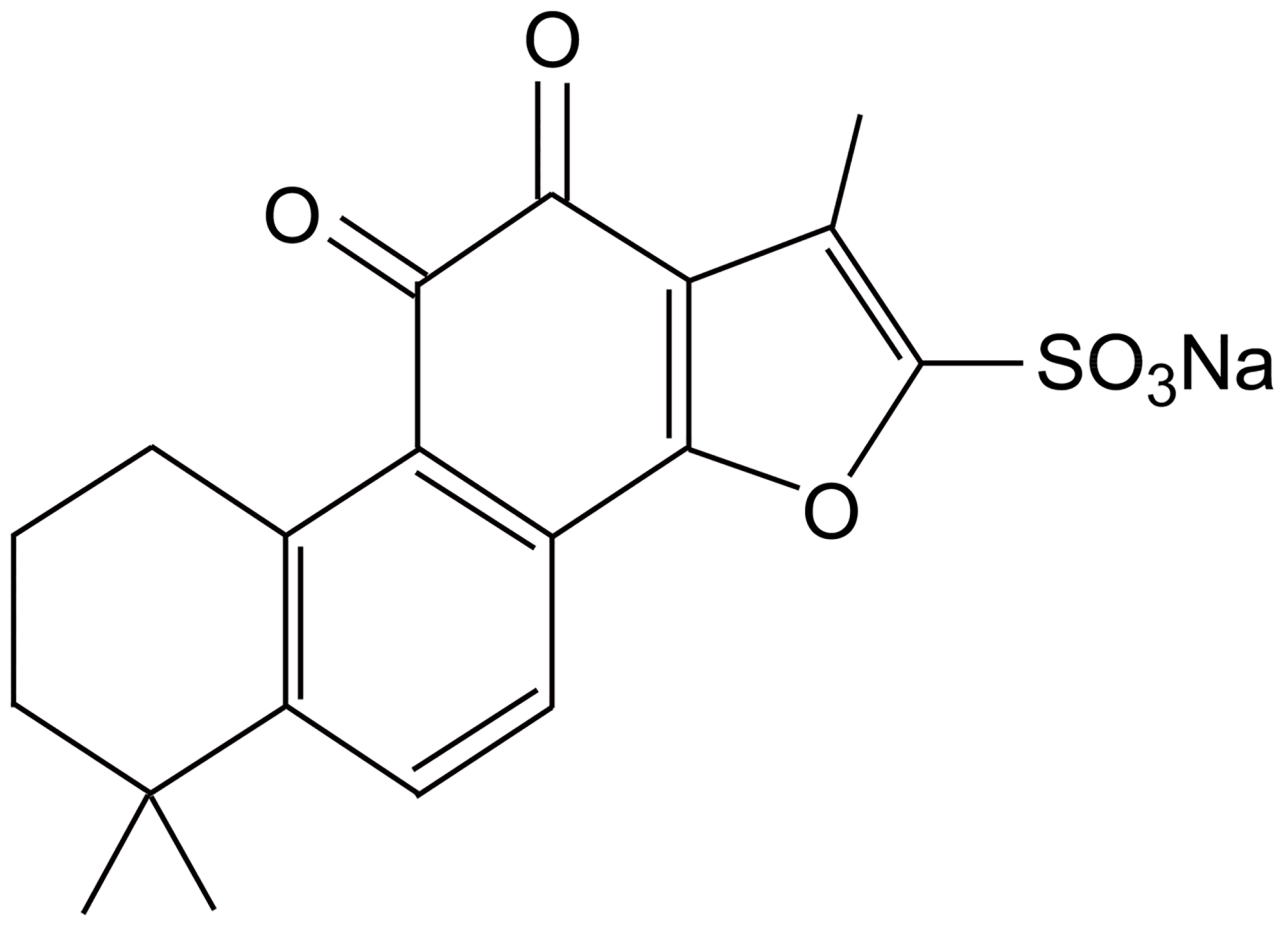
The chemical structure of STS.

Danshen, the dry root of *Salvia miltiorrhiza*, has been used for many years to prevent and cure cardiovascular diseases [12]. It is also a component of Chinese traditional lung-tonifying and expectorant decoction [13]. According to a clinical report, the aerosol inhalation of compound Danshen injection decreases the sputum viscosity and sputum elasticity of postoperative patients after pneumonectomy [14]. Since the viscosity and elasticity of sputum can be influenced by the hydration of ASL [15], the above finding suggests that some component of Danshen may regulate the hydration of ASL and improve the sputum discharge. The most abundant lipophilic compound in *Salvia miltiorrhiza* is tanshinone IIA, which can be transformed into sodium tanshinone IIA sulfonate (STS, Fig 1) by sulfonylation to acquire water solubility [16]. Moreover, STS shows similar pharmacological properties–the cardioprotective effects for instance, to Danshen [17–19]. Therefore, we propose that STS is able to regulate the ion transport of airway epithelium for the hydration of airway.

## Materials and methods

### Mouse

Male and female Kunming mice, each weighing 18–22 g, were obtained from the Experimental Animal Center of Guangdong Province. All mice were housed in environmentally controlled cages (21 ± 1°C, 12/12 h light/dark cycles) and had free access to food and water according to the Guidelines proclaimed by Sun Yat-Sen University Animal Use Committee (Guangzhou, China). For the preparation of trachea, mice were sacrificed by CO_2_ aspiration. All animal experiments were approved by Sun Yat-Sen University Animal Use Committee with the approval number No.0013121701 and No.0014022401.

### Measurement of short-circuit current (*I*_sc_)

Tracheas were cut longitudinally and mounted in Ussing chambers (Physiologic Instruments, San Diego, CA) with an inner area of 0.03 cm^2^. Both sides of the chamber were perfused with 6 ml Krebs-Henseleit (K-H) solution composed of (in mM): 117 NaCl, 4.7 KCl, 1.2 MgSO_4_, 24.8 NaHCO_3_, 1.2 KH_2_PO_4_, 2.5 CaCl_2_, and 11.1 glucose and aerated with 95% O_2_-5% CO_2_ at 37°C. The transepithelial potential difference of mouse trachea was clamped at 0 mV using VCC MC6 (Physiologic Instruments, San Diego, CA) to record *I*_sc_. The change of *I*_sc_ was recorded and analyzed by BL-420E+ system (Chengdu Technology & Market Co. Ltd, Chengdu, China). Changes in *I*_sc_ (Δ*I*_sc_) were defined as the difference between the value at baseline and that at a peak following compound addition. A positive *I*_sc_ value represents a net movement of anions from basolateral to apical side. For the measurement of *I*_sc_ under Cl^−^ free condition, Cl^−^ was replaced by gluconate.

### Primary culture of mouse tracheal epithelial cells

Mouse tracheas were placed in Ca^2+^ and Mg^2+^-free Hanks Balanced Salt Solution with 0.25% (w/v) trypsin at 4°C overnight. On the second day, tracheas were gently shaken in trypsin several times to make the surface epithelial cells fall off while the structure of tracheas remained largely intact so that other tracheal cells would not be collected. Afterwards, the tracheas were removed. The tracheal epithelial cells were collected by centrifugation (400 × g, 5 min). In the end, the immunostaining against keratin (dilution 1:150, keratin 27/28 (T-14), Santa Cruz, USA and dilution 1:100, FITC-rabbit anti-goat IgG, BOSTER, China) was used to identify the pureness of epithelial cells. Cells were cultured on coverslips in Keratinocyte-serum free medium (Gibco, USA) containing 100 U/ml penicillin and 100 mg/ml streptomycin (Hyclone, USA) for 1–2 days.

### Measurement of intracellular calcium

Mouse tracheal epithelial cells were loaded 5 μM fluo-3 AM in normal physiological saline solution (N-PSS) for 45 min following two washes with N-PSS which contained (in mM): 140 NaCl, 1 KCl, 1 MgCl_2_, 1 CaCl_2_, 5 HEPES and 10 glucose (pH 7.4). Then, cells were bathed in N-PSS or Ca^2+^-free physiological saline solution (Ca^2+^-free PSS) for the following experiments. In Ca^2+^-free PSS, CaCl_2_ was substituted by 2 mM EGTA. The image of fluorescence signal was performed by a laser scanning confocal imaging system (TCS SP2; Leica Microsystems, Wetzlar, Germany).

### Chemicals

Sodium Tanshinone IIA sulfonate (purity ≥ 98%, Lot: B1623119) was obtained from Aladdin (Shanghai, China). Tetrodotoxin (TTX), atropine, N-phenylanthranilic acid (DPC), 4,4’-diisothiocyanatostilbene-2,2’-disulfonate (DIDS), forskolin, CFTR_inh_172, MDL-12330A hydrochloride, 2-Aminoethoxydiphenyl borate (2-APB), thapsigargin, BAPTA-AM, U-73122 hydrate, capsazepine were purchased from Sigma-Aldrich (St. Louis, MO, USA). Tannic acid and all other general laboratory reagents were provided by Guangzhou Chemical Reagent Factory (Guangzhou, China).

### Statistical analysis

Values were presented as mean ± S.E.M. (*n* represents the number of experiments). Statistical analyses were conducted using Origin 8.0 software (OriginLab Corporation, Northampton, USA). Statistical significance was evaluated by unpaired Student’s t-test or analysis of variance (ANOVA) followed by Bonferroni correction for multiple comparison. Significantly different values (*P* < 0.05) are marked with asterisks (*).

## Results

### The *I*_*sc*_ response induced by STS in mouse tracheal epithelium

**Fig 2:**
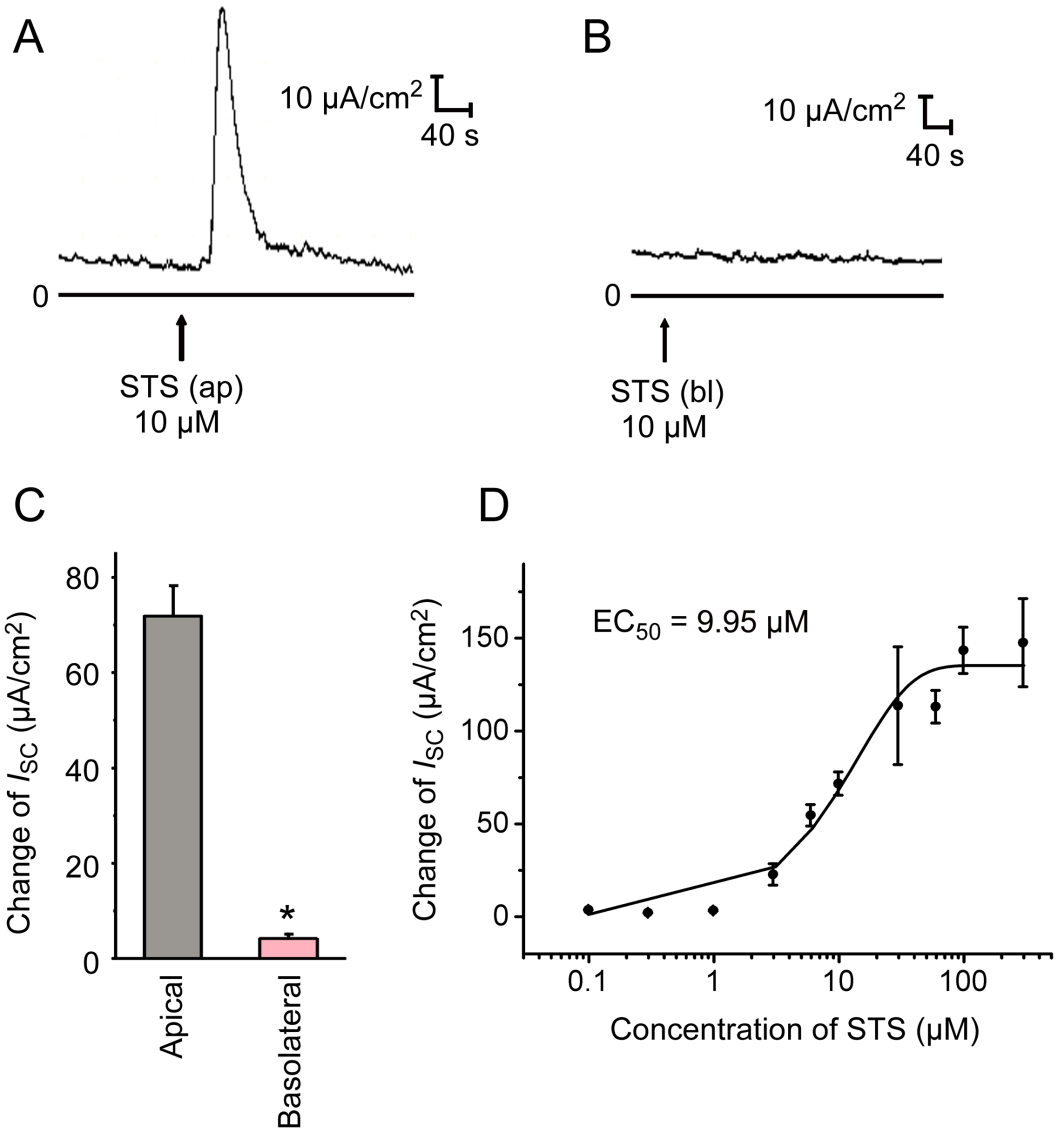
Effect of STS on *I*_*sc*_ response in mouse tracheal epithelium. (A) Apical application (ap) of 10 μM STS induced a fast and transient increase in *I*_*sc*_, as visible in the typical current trace. (B) Typical *I*_*sc*_ response of basolateral application (bl) of 10 μM STS in normal K-H solution. (C) Comparison of Δ*I*_sc_ induced by STS in apical (*n* = 9) and basolateral (*n* = 5) in mouse tracheal epithelium. Values are mean ± S.E.M. (Student’s t-test, *P < 0.05 compared with the apical group). (D) Dose-response curve of the apical application of STS-induced changes on *I*_*sc*_. The concentrations of STS are 0.1, 0.3, 1, 3, 6, 10, 30, 60, 100 and 300 μM. Each data point is mean ± S.E.M. (*n* = 3–6). EC_50_: effective concentration for half-maximal Δ*I*_*sc*_ after apical STS application. The arrows mark the time at which the drugs were added.

Application of STS (10 μM) to the apical side of the mouse tracheal epithelium caused a sharp increase in *I*_*sc*_ (Fig 2A and 2C, Δ*I*_*sc*_ = 71.9 ± 6.2 μA/cm^2^, n = 9). Yet, basolateral application of STS (10 μM) induced only a slight change of *I*_sc_ (Fig 2B and 2C, Δ*I*_sc_ = 4.2 ± 0.9 μA/cm^2^, n = 6). The diffusion rate of STS in the chamber and manually labeling in record software may cause the deviation of time points of STS application showed in different experiments. Moreover, STS-induced *I*_sc_ at the apical side of mouse tracheal epithelium was dose-dependent with an EC_50_ of 9.95 μM (Fig 2D).

### STS induced Cl^−^ secretion in mouse tracheal epithelium

**Fig 3:**
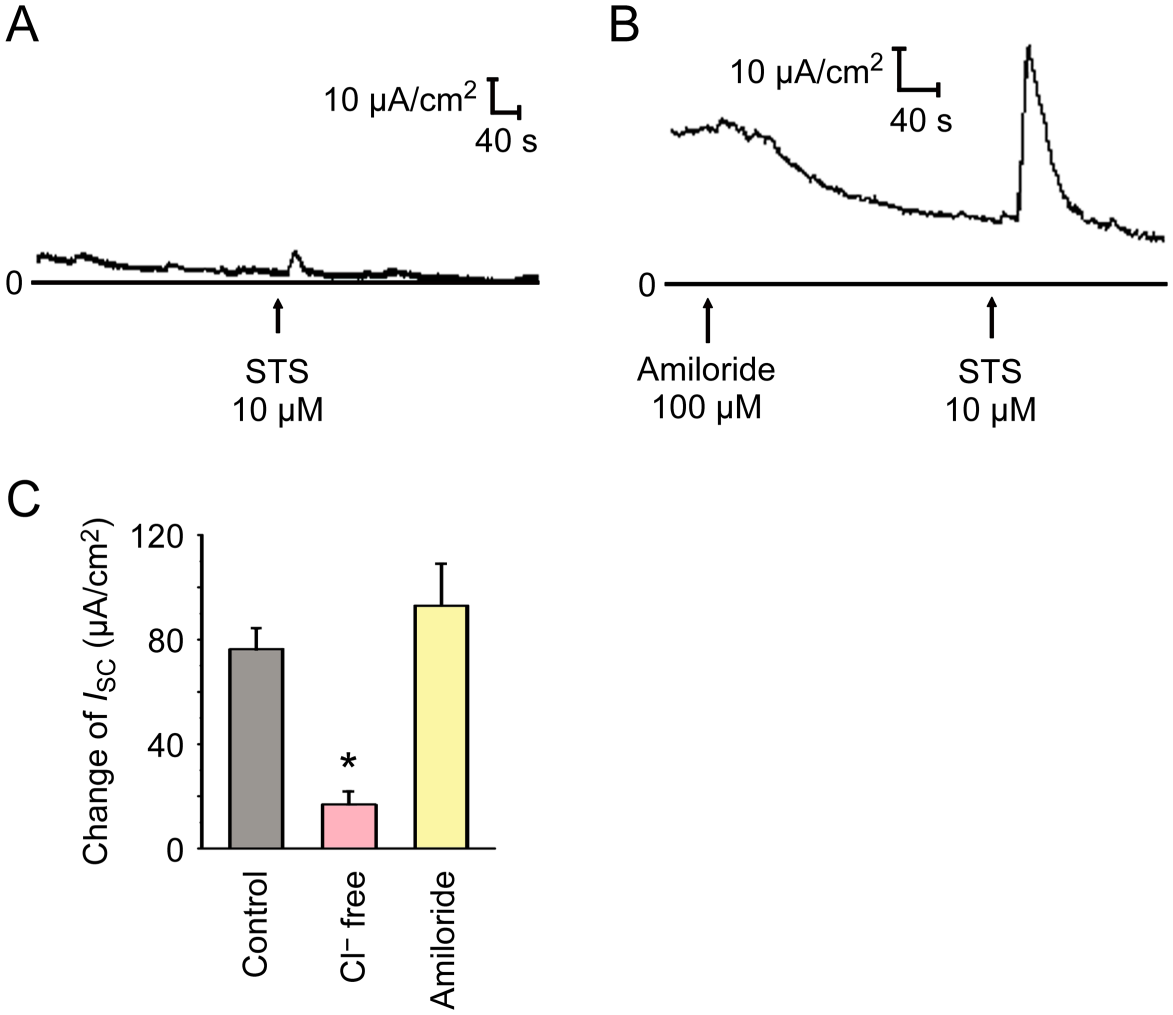
Cl^−^ dependence of the *I*_sc_ response to STS. (A) Representative recording of the *I*_sc_ activated by STS (10 μM, apical) in Cl^−^ free K-H solution (*n* = 6). (B) Representative curve of the *I*_sc_ activated by STS (10 μM, apical) in K-H solution pretreated with amiloride (100 μM, apical, *n* = 5). (C) Comparison of STS (10 μM, apical, *n* = 11) induced Δ*I*_sc_ obtained in Cl^−^ free K-H solution and normal K-H solution with or without amiloride (100 μM, apical). Values are mean ± S.E.M. (ANOVA, *P < 0.05 compared with the control). The arrows mark the time at which the drugs were added.

The increase of *I*_sc_ can be induced by cation absorption to basement membrane, anion secretion to lumen or the combination of both. To demonstrate whether Cl^−^ was involved in the STS-induced *I*_*sc*_, mouse trachea was bathed in Cl^−^-free K-H solution for 15–20 min until the current was stable. We found that removing ambient Cl^−^ abolished the STS-induced *I*_sc_ response (Fig 3A and 3C). The epithelial Na^+^ channel (ENaC) is involved in cation absorption of airway epithelium [3]. Application of the ENaC blocker amiloride (100 μM) to the apical side caused a decrease of basal *I*_sc_, but the STS-induced *I*_sc_ in the presence of amiloride was not significantly altered (Fig 3B and 3C), suggesting that the STS-induced *I*_*sc*_ is Na^+^ independent. In brief, STS-induced *I*_sc_ is primarily a Cl^−^ current.

### STS activated CaCC

**Fig 4:**
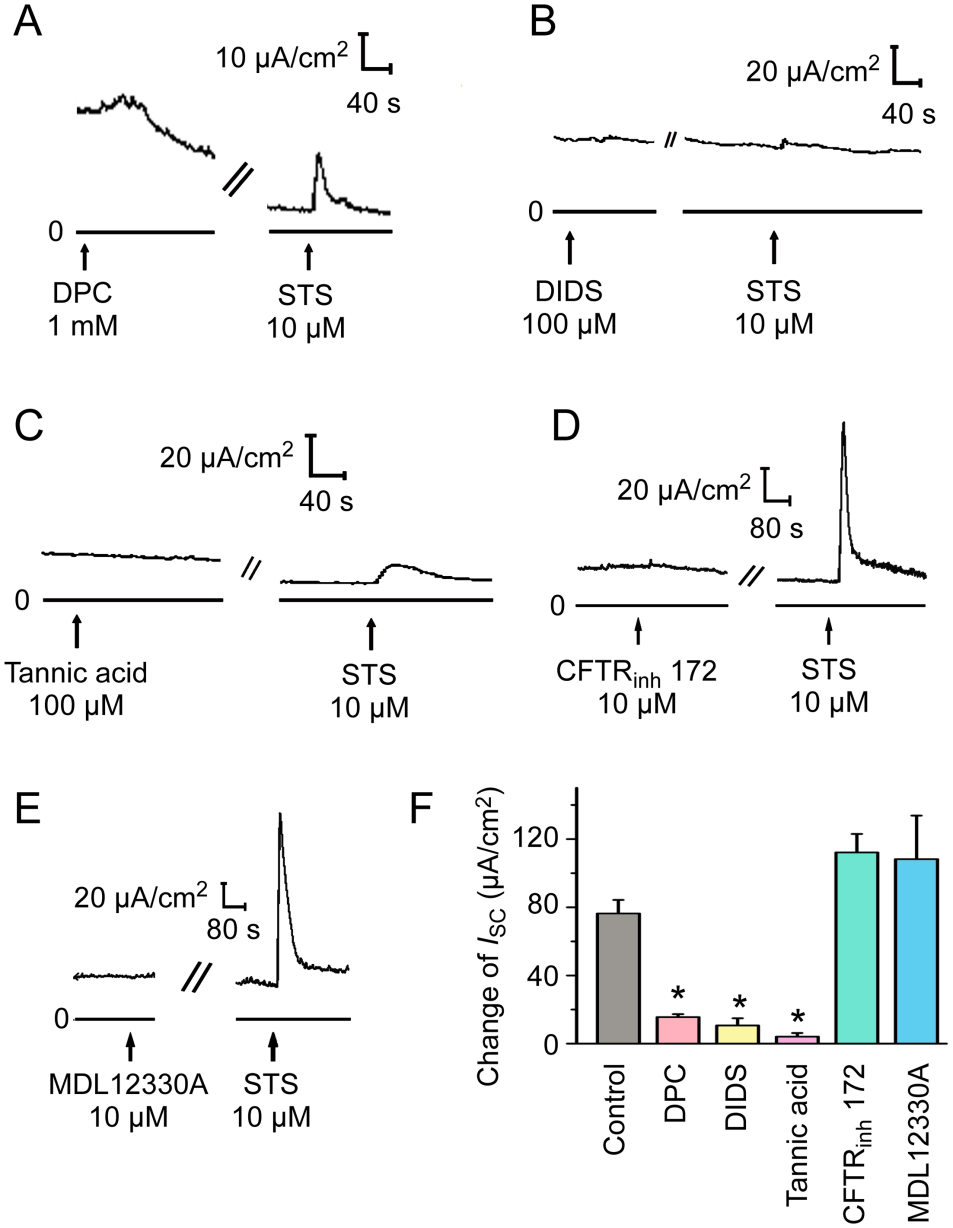
Effect of different Cl^−^ channel blockers on *I*_*sc*_ induced by STS. Representative recordings of *I*_sc_ induced by STS (10 μM, apical) pretreated with (A) the non-specific Cl^−^ channel blocker DPC (1 mM, apical, *n* = 6), (B) the Ca^2+^-activated Cl^−^ channel (CaCC) blocker DIDS (100 μM, apical, *n* = 6), (C) tannic acid (100 μM, apical, *n* = 6), (D) the CFTR blocker CFTR_inh_172 (10 μM, apical, *n* = 6), (E) the adenylate cyclase inhibitor MDL-12330A (10 μM, apical, *n* = 5), for 15 min. (F) Comparison of the effects of different Cl^−^ channel blockers and MDL-12330A on STS (10 μM, apical) induced Δ*I*_sc_. Values are mean ± S.E.M. (ANOVA, *P < 0.05 compared with the control). The arrows mark the time at which the drugs were added.

To investigate which Cl^−^ channel is involved in the STS-induced *I*_*sc*_ response, different Cl^−^ channel blockers were used. Application of the non-specific Cl^−^ channel blocker DPC (1 mM) or the CaCC blockers DIDS (100 μM) and tannic acid (100 μM) significantly reduced the STS-induced *I*_*sc*_ response (Fig 4A–4C, *n* = 6, *P* < 0.05). On the other hand, neither CFTR_inh_ 172 (10 μM), the specific CFTR blocker, nor MDL-12330A (10 μM), the adenylate cyclase inhibitor, had significant effects on the *I*_*sc*_ induced by STS (Fig 4D and 4E), suggesting that CaCC, but not CFTR, was involved in the Cl^−^ secretion induced by STS.

### STS-induced *I*_sc_ response was dependent on muscarinic acetylcholine receptor on mouse tracheal epithelium

**Fig 5:**
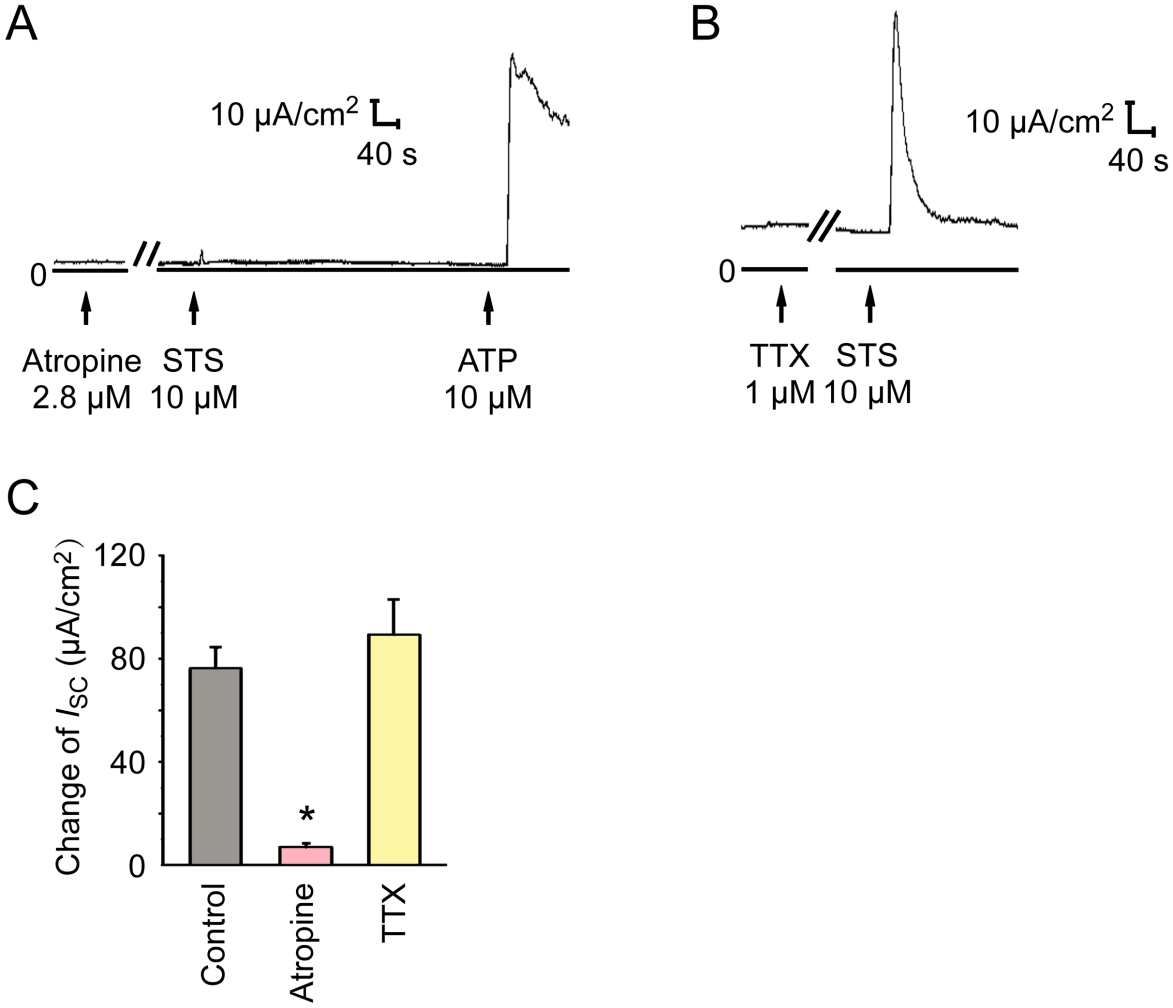
Effect of atropine and Tetrodotoxin (TTX) on apical-applied STS-induced *I*_*sc*_. (A) The effect of 2.8 μM atropine on apical applied STS-induced *I*_sc_. ATP was applied as a positive control to check the activity of mouse tracheal epithelium. (B) Apical application of 10 μM STS-induced *I*_*sc*_ response in the presence of 1 μM TTX. (C) Atropine (2.8 μM, apical, *n* = 7) inhibited STS-induced *I*_sc_ response significantly. In the presence of TTX (1 μM, basolateral, *n* = 8), Δ*I*_sc_ induced by STS was not significantly altered compared to the control (10 μM, apical, *n* = 11). Values are mean ± S.E.M. (ANOVA, *P < 0.05 compared with the control). The arrows mark the time at which the drugs were added.

It has been reported that CaCC can be activated by muscarinic acetylcholine receptor (mAChR) in mouse airway epithelium [20]. To study the role of mAChR in STS-induced *I*_*sc*_, the apical side of mouse tracheal epithelium was pretreated with atropine (2.8 μM), an mAChR antagonist, followed by administration of STS (10 μM). Our results showed that STS-induced *I*_sc_ response was inhibited by atropine (Fig 5A and 5C), indicating that mAChR mediated the effect of STS on ion transport of mouse tracheal epithelium.

Airway secretion of the isolated trachea can be influenced by intrinsic airway neurons. Activation of neurons in the intrinsic nerve net by noxious stimuli caused an immediate increase in short circuit current [21, 22]. Thus, we supposed that STS might activate the intrinsic acetylcholine (ACh)-containing airway neurons, which release ACh to the periphery of airway epithelium, leading to activating the mAChR and CaCC of airway epithelium. We conducted experiments with TTX (1 μM), a selective neuronal Na^+^ channel blocker that could block conducted action potentials in intrinsic airway nerve net. However, no statistical significant difference was observed (Fig 5B and 5C) in the STS-induced *I*_*sc*_ with TTX, suggesting that the intrinsic airway neurons were not involved.

ACh is an endogenous ligand of mAChR. Several research show that chemosensory cells scatter within mouse trachea and release ACh via activation of bitter taste receptor-Transient receptor potential channel M5 (TRPM5) transduction pathway [23, 24]. Notably, Danshen tastes bitter according to the traditional Chinese medicine theory [25]. Thus, we interrogated whether STS induced mAChR-dependent Cl^−^ secretion by activating bitter taste receptor-TRPM5 pathway in tracheal chemosensory cells, which causes the release of ACh and the following Cl^−^ secretion. However, simultaneous application of 1 mM quinidine, a blocker of TRPM5 [26], at both apical and basolateral sides of mouse trachea did not significantly inhibit the effect of STS (S1 Fig), suggesting that STS-stimulated mouse tracheal Cl^−^ secretion might not be mediated by bitter taste receptor-TRPM5 pathway.

### The involvement of Ca^2+^ signaling in STS-induced Cl^−^ secretion in mouse tracheal epithelium

**Fig 6:**
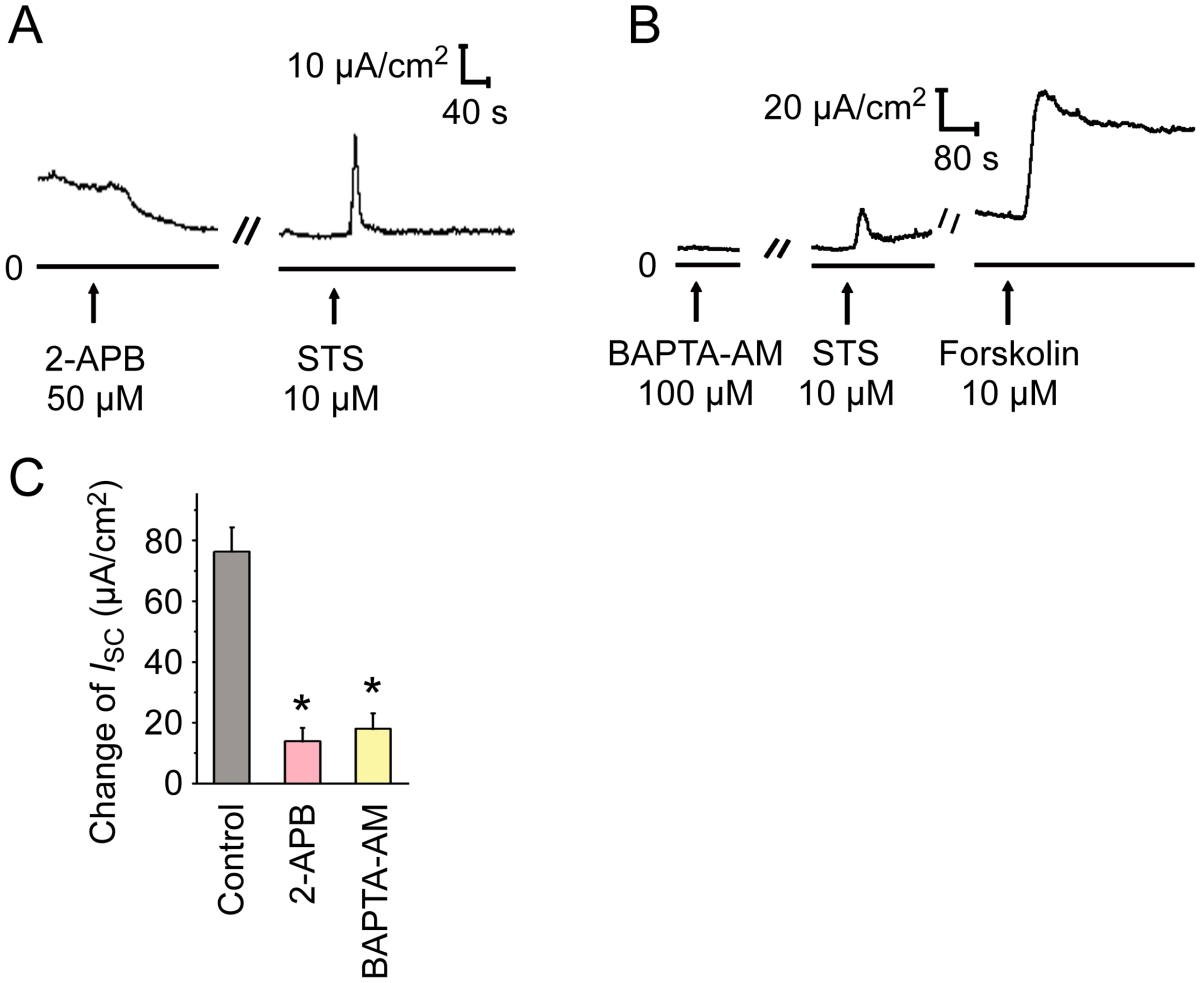
Effect of Ca^2+^ signaling inhibitors on response to STS. Representative current traces of *I*_sc_ induced by STS (10 μM, apical) pretreated with (A) the IP_3_ receptor antagonist 2-APB (50 μM, apical, *n* = 6), (B) the intracellular Ca^2+^ chelator BAPTA-AM (100 μM, apical, *n* = 5). (C) Comparison of the effects of different Ca^2+^ signaling inhibitors on STS (10 μM, apical) induced Δ*I*_sc_. Values are mean ± S.E.M. (ANOVA, *P < 0.05 compared with the control). The arrows mark the time at which the drugs were added.

In light that an increase in intracellular Ca^2+^ concentration ([Ca^2+^]_i_) leads to activation of CaCC [27], we supposed that STS might increase [Ca^2+^]_i_ after the activation of mAChR, ultimately activating CaCC. 2-APB, an antagonist of inositol 1,4,5-triphosphate (IP_3_) receptor, led to a current decrease and inhibited the Cl^−^ secretion induced by STS (Fig 6A and 6C). Pretreatment of BAPTA-AM, an intracellular Ca^2+^ chelator, blocked the *I*_sc_ induced by STS, and 10 μM forskolin (adenylate cyclase agonist), which can activate CFTR by increasing the intracellular level of cAMP was used as a positive control (Fig 6B and 6C). The effect of these inhibitors indicated that the mAChR-mediated Ca^2+^ signaling was involved in the STS-induced Cl^−^ secretion in mouse tracheal epithelium.

**Fig 7:**
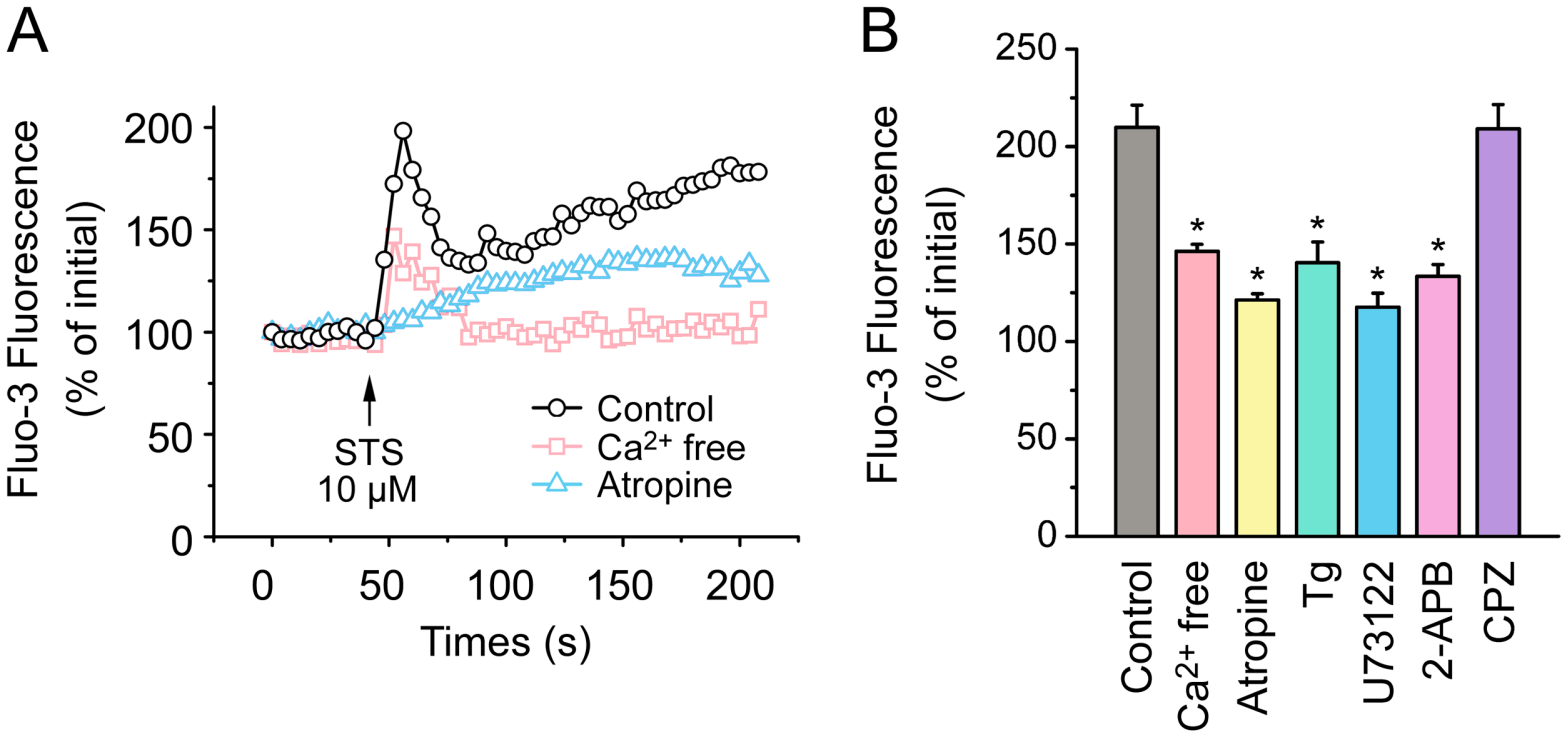
Effects of STS on intracellular Ca^2+^ in mouse airway epithelial cells. (A) Representative intracellular Ca^2+^ transients elicited by STS (10 μM) detected by fluo-3 fluorescence in the presence or absence of 2.8 μM atropine or in extracellular Ca^2+^ free solution. (B) Statistical analysis of intracellular Ca^2+^ in response to STS in N-PSS (control, 10 μM, *n* = 13) or in Ca^2+^ free PSS (*n* = 5) and the inhibitory effects of atropine (2.8 μM, *n* = 12), U73122 (20 μM, *n* = 3), 2-APB (50 μM, *n* = 6), Tg (10 μM, *n* = 7) and CPZ (10 μM, *n* = 3) on the Ca^2+^ transients elicited by STS (10 μM). Data is expressed as a percentage of the initial fluo-3 fluorescence. Values are mean ± S.E.M. (ANOVA, *P < 0.05 compared with the control).

To further confirm the critical role of Ca^2+^, fluo-3 Ca^2+^ imaging was performed using primary cultured mouse tracheal epithelial cells. Treatment with 10 μM STS resulted in an increase in [Ca^2+^]_i_ which consisted of a transient peak followed by a plateau phase (Fig 7A). However, both values of the response at peak and plateau phase decreased when STS was applied in the presence of atropine (2.8 μM, Fig 7A), confirming the crucial role of mAChR in STS-induced elevation in [Ca^2+^]_i_. Phospholipase C (PLC), IP_3_ receptor, endoplasmic reticulum (ER) Ca^2+^ stores are key elements for the Ca^2+^ release caused by mAChR activation. A series of inhibitors including U73122 (20 μM, an inhibitor of PLC), 2-APB (50 μM) and thapsigargin (Tg, 10 μM, the inhibitor of ER Ca^2+^-ATPases) were used respectively and the peak values of the responses induced by STS (Fig 7B) in presence of these inhibitors were reduced. In addition, when the extracellular Ca^2+^ was removed by perfusion with Ca^2+^-free solution, STS caused a reduced transient peak with a lower level of plateau phase compared with the control (Fig 7A), indicating that Ca^2+^ influx also contributed to the STS-induced elevation in [Ca^2+^]_i_. Finally, we tested whether transient receptor potential vanilloid 1 (TRPV1), a cation-permeable Ca^2+^ channel, mediated the Ca^2+^ influx in the STS-induced elevation in [Ca^2+^]_i_. But the results showed that the STS-induced response was not decreased by application of the TRPV1 blocker capsazepine (CPZ, 10 μM, Fig 7B). The individual data points behind means, medians and variance measures presented in the results are available in S1 Dataset.

## Discussion

Currently, various studies have been done regarding the effects of STS on treatment of cardiovascular disease [16]. In addition, several ion channels are modulated by STS and its precursor Tanshinone IIA in some cardiovascular disease models, such as human cardiac KCNQ1/KCNE1 (I-Ks) K^+^ channels [28], high conductance Ca^2+^ activated K^+^ channels (BK_Ca_) [29], K_V_2.1 and K_V_1.5 [30]. In this research, we have, for the first time, observed that an apical application of STS activated CaCC in an mAChR-dependent way and induced Cl^−^ secretion in mouse tracheal epithelium, indicating that STS might regulate Cl^−^ transport in the airway.

The depth of airway surface liquid is contributed by the balance between Na^+^ absorption and Cl^−^ secretion [31, 32]. ENaC mediates Na^+^ absorption across the apical plasma membrane while CFTR and CaCC mediated the active Cl^−^ secretion. CFTR is a cAMP–activated Cl^−^ channel and CaCC is activated by an increase in [Ca^2+^]_i_ [3, 27]. We observed the high baseline currents in Figs 3B, 4A and 4B, and application of amiloride or DPC caused the rundown of baseline currents, indicating ENaC and Cl^−^ channels are involved in the basal currents. The magnitude of initial background current is determined by initial transepithelial voltage potential (Vt) and series resistance of the circuit in voltage clamp equipment plus mouse tracheal mucosa. According to Ohm’s Law, a high Vt or a low resistance can cause a high current. Robust basal Na^+^ absorption and Cl^−^ secretion could lead to a high initial Vt, while the damage of tight junction during mucosal preparation or some deviations in voltage clamp equipment decreases the series resistance, resulting in a general baseline shift. Thus, high initial background current in Figs 3B and 4A & 4B might be caused by the above reasons.

Few studies have investigated the effect of *Salvia miltiorrhiza* on Cl^−^ channel. The extract from the root of *Salvia miltiorrhiza* is reported to induce salivary fluid secretion by activating Na^+^-K^+^-Cl^−^ cotransporter (NKCC) to maintain Cl^−^ secretion, however, the involvement of Cl^−^ channel has not been investigated in the research [33]. In our study, STS induced a DIDS- and tannic acid-sensitive Cl^−^ secretion in mouse tracheal epithelium, which was not influenced by amiloride, CFTR_inh_172 or MDL-12330A (Fig 4). Meanwhile, either inhibiting the increase of [Ca^2+^]_i_ via 2-APB or chelating intracellular Ca^2+^ by BAPTA-AM abated STS-induced Cl^−^ secretion (Fig 6). Additionally, the Ca^2+^ image showed that STS increased [Ca^2+^]_i_ in primary cultured mouse tracheal epithelial cells through Ca^2+^ release and Ca^2+^ influx (Fig 7). All the results above indicated that STS could activate CaCC to mediate the *I*_sc_ response.

In salivary glands, the extract from the root of *Salvia miltiorrhiza* induced fluid secretion requires extracellular Ca^2+^ mediated increase in [Ca^2+^]_i_ and is not dependent on mAChR [33]. But we found that atropine was capable of blocking the Cl^−^ secretion induced by STS in mouse trachea (Fig 5A), indicating that mAChR was involved in the effect of STS. Therefore, we suppose that the difference in mechanisms underlying Cl^−^ secretion stimulated by extract of *Salvia miltiorrhiza* and STS might be caused by the comprehensive effects of extract of *Salvia miltiorrhiza* and the difference between salivary gland and airway. Cholinergic airway intrinsic neurons innervate airway smooth muscle and submucosal gland [34], so these neurons might stimulate airway epithelium Cl^−^ transport through the release of ACh. However, application of TTX to block the generation of action potential in airway intrinsic neurons showed that the effect of STS was not influenced by the pretreatment of TTX (Fig 5B). In addition, after application of STS to the primary cultured mouse airway epithelial cells, a preparation that is devoid of neurons, we observed an mAChR-dependent increase of [Ca^2+^]_i_ (Fig 7A). Thus, we concluded that STS was sensed by airway epithelial cells instead of airway intrinsic neurons.

The conserved Asp 103^3.32^ of mAChR serves as the key residue that binds to the cationic amine of mAChR agonist [35]. However, no cationic amine structure was observed at STS, indicating that STS may not be able to bind to mAChR like ACh. Meanwhile, both apical and basolateral membranes of mouse airway epithelia have functional mAChR [20], whilst STS only gave rise to the response at apical membrane, suggesting that STS might not bind to mAChR directly.

Another possibility is that STS provokes neuron-independent release of ACh in tracheal epithelium. According to the bitter taste property of STS and the presence of chemosensory cells with bitter taste transduction in mouse tracheal epithelium, we speculate STS might activate taste receptor type 2 (Tas2Rs), which are bitter taste receptors, and as a result subsequently induce Ca^2+^-dependent activation of TRPM5 followed by ACh release and thus influence Cl^−^ secretion of adjacent airway secretory cell. However, our data showed that 1 mM quinidine, the blocker of TRPM5 channel, did not significantly inhibit STS-induced *I*_sc_ (S1 Fig), suggesting that bitter taste transduction of mouse tracheal chemosensory cells were not involved in STS-induced Cl^−^ secretion. More studies should be done to find out the receptor of STS in airway epithelium.

It is reported that mAChR exist as five subtypes, M1-5. ACh induced Cl^−^ secretion in mouse airway epithelium is mainly mediated by M3 [20]. The activation of M3 mAChR activates phospholipase C (PLC) which mediates the hydrolysis of phosphatidylinositol 4,5-bisphosphate (PI(4,5)P_2_) to generate inositol 1,4,5-triphosphate (IP_3_) and diacylglycerol (DAG). Then, IP_3_ binds to the IP_3_ receptor at ER and releases the Ca^2+^ to the cytoplasm [36]. Our findings showed that U73122, 2-APB and Tg reduced the increase of STS-induced [Ca^2+^]_i_ (Fig 7B). In Ca^2+^-free solution, we observed that the transient peak caused by STS was partially reduced and plateau phase was almost fully abolished (Fig 7A), indicating that extracellular Ca^2+^ influx contributed to the plateau phase of STS-induced elevation of [Ca^2+^]_i_. We supposed that the partially reduced transient peak might result from a slow discharging in intracellular Ca^2+^ stores when the cells were surrounded by Ca^2+^-free solution. Functional expression of TRPV1 was found in bronchial epithelial cells and sensory neurons of human and mouse [37, 38]. TRPV1 is a Ca^2+^ permeable cation channel activated by noxious heat and some chemical irritants. We hypothesized whether TRPV1 could detect STS as a noxious irritant and mediate Ca^2+^ influx. However, capsazepine did not influence the STS-induced [Ca^2+^]_i_ increase (Fig 7B), indicating TRPV1 might be not involved. However, capsazepine, a competitive TRPV1 antagonist, is a lipophilic molecule which passes through the plasma membrane to block the intracellular “vanilloid-pocket” domain of TRPV1 [39, 40]. Our data did not exclude that STS might act on TRPV1 independently from capsazepine inhibition via another binding site. In summary, our data showed that STS activated mAChR of airway epithelial cells indirectly and induced [Ca^2+^]_i_ increase via both Ca^2+^ store release and Ca^2+^ influx.

Activation of CaCC by ATP or UTP released from airway secretory cells contributes to the optimized depth of the periciliary layer [41]. The hydration of airway surface promotes the mucus clearance and ultimately benefits the airway defense against bacterial infection [4]. Previously, Danshen has been used to treat dry mouth and dry eyes, both of which have symptoms associated with the reduction of fluid secretion [33]. However, its effect on airway liquid secretion has not been studied thoroughly. Our current findings suggested STS might play a role in airway hydration and innate immunity of airway epithelium via CaCC. In recent years, transmembrane protein 16A (TMEM16A) has been identified as CaCC channel molecule. However, TMEM16A was involved in the pathogenesis of mucus hypersecretion induced by the T helper type 2 (T_h_2) cytokine interleukin-13 [42, 43]. Thus, further animal model and *in vivo* experiments are needed to investigate whether STS could ameliorate the airway dehydration symptom in cystic fibrosis and COPD. The effect of STS on airway epithelium in T_h_2 cytokines environment also needs to be considered carefully.

Chinese herbal monomers possess many advantages, such as good quality, controllability and clear toxicology. Conclusively, this study demonstrated that STS induced Cl^−^ secretion via CaCC in an mAChR-dependent way in airway epithelium, which suggested a new application field of this traditional Chinese medicine monomer in respiratory system.

## Supporting information

S1 Fig Effect of quinidine on apical-applied STS-induced *I*_*sc*_. Comparison of the effects of application of 1 mM quinidine at both apical and basolateral side of mouse trachea on STS (10 μM, apical) induced Δ*I*_sc_. Δ*I*_sc_ induced by STS (10 mM, apical) with the application of quinidine is 69.5 ± 13.7 μA/cm^2^ (*n* = 4). Δ*I*_sc_ caused by STS (10 mM, apical) without quinidine is 71.9 ± 6.2 μA/cm^2^ (*n* = 9). Values are mean ± S.E.M. (Student’s t-test, NS, no significant difference was observed).
(TIF)

S1 Dataset The individual data points behind means, medians and variance measures presented in the results. (XLSX)
