# Supplementary figures and images for: Sodium tanshinone IIA sulfonate stimulated Cl− secretion in mouse trachea
Source: PLoS One. 2017 May 22;12(5):e0178226. doi: 10.1371/journal.pone.0178226 (PMC5440052; doi:10.1371/journal.pone.0178226)

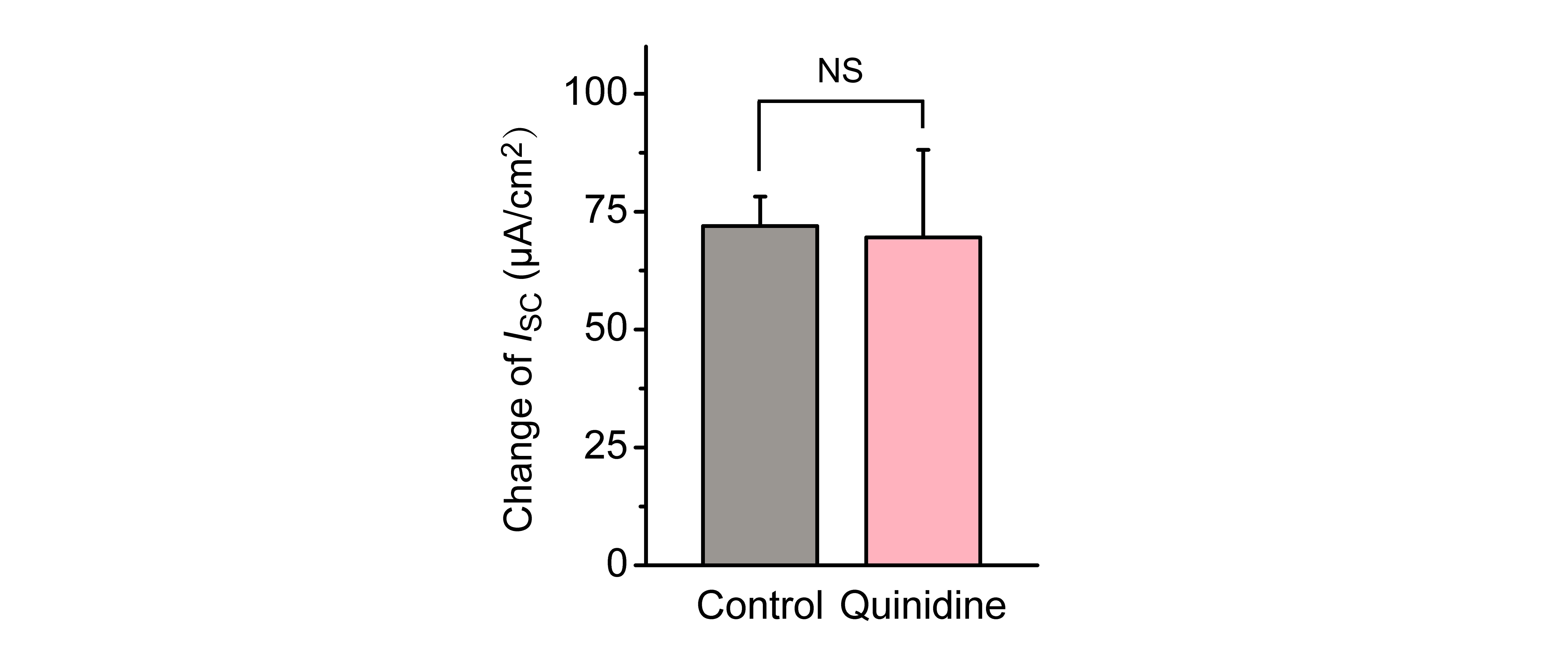

Supplement: S1 Fig — Comparison of the effects of application of 1 mM quinidine at both apical and basolateral side of mouse trachea on STS (10 μM, apical) induced ΔIsc. ΔIsc induced by STS (10 mM, apical) with the application of quinidine is 69.5 ± 13.7 μA/cm2 (n = 4). ΔIsc caused by STS (10 mM, apical) without quinidine is 71.9 ± 6.2 μA/cm2 (n = 9). Values are mean ± S.E.M. (Student’s t-test, NS, no significant difference was observed). (TIF) [file pone.0178226.s001.tif]
